# Older patients with non-ST-elevation myocardial infarction: which treatment strategies do we currently use?

**DOI:** 10.1007/s12471-023-01842-8

**Published:** 2024-01-03

**Authors:** Kirsten Boerlage-van Dijk

**Affiliations:** Department of Cardiology, Jeroen Bosch Ziekenhuis, ’s-Hertogenbosch, The Netherlands

With the elderly population growing rapidly, registries like the nationwide POPular AGE registry will become increasingly important for gaining more insight into our current treatment strategies in elderly patients with non-ST-elevation myocardial infarction (NSTEMI). One in four persons in Europe and North America is expected to be ≥ 65 years old by 2050, according to data from the United Nations [1]. This will lead to an increase in older patients with cardiovascular disease in the decades to come. Scientific data on older persons, especially those with comorbidities and/or frailty, are unfortunately relatively scarce. In this issue of the *Netherlands Heart Journal*, Gimbel et al. nicely report the results of their nationwide POPular AGE registry [2].

In the recent NSTEMI guideline from the European Society of Cardiology (ESC), it is recommended that the same diagnostic and interventional strategies be used for older patients as are being used for younger patients [3]. Gimbel et al. have shown that a high percentage (75%) of older patients with NSTEMI in their cohort underwent coronary angiography, but only 51% of these patients underwent revascularisation (40% percutaneous coronary intervention and 11% coronary artery bypass grafting), although 88% of these patients had a significant coronary lesion. The authors suggest that a conservative strategy is often preferred because this elderly population is considered to be at too high a risk of complications with an invasive treatment strategy. However, Gimbel et al. demonstrated a significantly higher event rate in the pharmacologically treated patients compared to the patients who underwent revascularisation. This finding is comparable to the results of a recent meta-analysis performed by Khalil et al. on an invasive versus a conservative strategy in older patients with NSTEMI. This meta-analysis demonstrated a significantly lower risk of major adverse cardiac and cerebrovascular events in older patients with NSTEMI in whom an invasive strategy was used than in patients managed by a conservative strategy [4]. It would be interesting to know why these patients did not undergo revascularisation in the POPular AGE registry: due to high-risk lesions, because they were considered to be too frail, due to comorbidities which excluded them from revascularisation or because the patients refused an invasive strategy? Unfortunately, the authors do not present the reasons for not performing an intervention.

With ageing of the population, there will be a higher prevalence of frailty among the elderly. Frailty in patients with NSTEMI is associated with a greater risk of complications in both medical therapies and cardiac interventions and is associated with a worse prognosis [5]. The ESC guideline states that data on older frail patients with NSTEMI are lacking and recommends risk stratification in these patients as well as offering medical therapy and an invasive strategy in patients with a high risk of future cardiovascular events and a low risk of complications [3]. Recently, the results of the (to date) only randomised clinical trial (the MOSCA-FRAIL trial) to investigate an invasive versus a conservative strategy in older frail patients with NSTEMI were published. This trial demonstrated that there was no benefit to using an invasive strategy as regards survival. The authors recommend medical therapy and watchful observation for older frail patients with NSTEMI [6]. Future trials and registries concerning the older population with NSTEMI, including frail patients, are warranted.

Another issue that would be interesting to investigate in the older population with NSTEMI is quality of life after the different treatment strategies. Older patients might have other goals and expectations of care and treatment than younger patients, and quality of life after a specific treatment strategy might be a more important issue for older patients than survival.

The authors of the POPular AGE registry also demonstrated the use of antithrombotic therapy in their cohort. At discharge 56.7% of the patients used dual antiplatelet therapy (DAPT) and 27.4% used oral anticoagulation (OAC) with at least one P2Y12 inhibitor. Ten percent of the patients were discharged on triple therapy ((N)OAC + aspirin + P2Y12 inhibitor). There was a relatively low major bleeding risk in the POPular AGE registry of 3.9% at 1‑year follow-up. According to the authors, this might be explained by early discontinuation of the triple therapy at 30 days and be due to discontinuation of one antithrombotic agent when moderate to severe bleeding occurred. Most of the patients who received (N)OAC used clopidogrel instead of the other more potent P2Y12 inhibitors, in accordance with our current ESC guidelines [3]. The authors did not comment on how many patients used clopidogrel rather than one of the more potent P2Y12 inhibitors when they were discharged on DAPT, but only mentioned clopidogrel use in patients who received OAC. In the POPular AGE trial, Gimbel et al. showed that in older patients with NSTEMI there were fewer bleeding events in patients that used clopidogrel compared to those taking ticagrelor [7]. It would be interesting to know what choice the cardiologists made for their patients and if this might be an explanation for the relative low bleeding risk in this registry.

In summary, this nationwide registry gives a good reflection of the treatment strategies in older patients with NSTEMI. Hopefully more registries and trials in this older NSTEMI population will follow, with some additional endpoints that are especially important for the older population, to provide more data for specific recommendations for the rapidly growing elderly population.
